# Increased occurrence of *Staphylococcus aureus* in wild ungulates intended for human consumption – An update on the monitoring in Brandenburg, Germany

**DOI:** 10.1016/j.onehlt.2025.101286

**Published:** 2025-11-25

**Authors:** Tobias Lienen, Anneluise Mader, Maciej Durkalec, Martin H. Richter, Sven Maurischat

**Affiliations:** a German Federal Institute for Risk Assessment (BfR), Department Biological Safety, 10589 Berlin, Germany; b German Federal Institute for Risk Assessment (BfR), Department Safety in the Food Chain, 10589 Berlin, Germany

**Keywords:** *Staphylococcus aureus*, Monitoring, Wildlife reservoirs, Game animals, Antimicrobial resistance, Foodborne pathogens

## Abstract

Monitoring data on *Staphylococcus* (*S*.) *aureus* in wild animals are scarce, yet transmission to humans and livestock remains a concern. *S. aureus* can cause infections in humans and animals, and the production of staphylococcal enterotoxins (SEs) may cause food poisoning. This study monitored the occurrence of *S. aureus* in wild ungulates intended for human consumption in Brandenburg, Germany.

A total of 323 nasal swabs were collected from wild boar, roe, fallow, and red deer during the 2023/24 hunting season. Samples were analyzed by selective enrichment. Isolates were identified using MALDI-TOF MS. Whole-genome sequencing was used for analyses of genotypes, antimicrobial resistance (AMR) and virulence genes as well as phylogeny. Phenotypic AMR was also determined.

*S. aureus* was recovered from 58 swabs (18 %), exceeding the occurrence in 2021/22 (8 %) by over twofold. Fallow deer showed the highest detection rate (39 %), followed by red deer (32 %), roe deer (16 %), and wild boar (10 %). *S. aureus* isolates represented diverse sequence types (STs), with a majority of ST1, ST133 and ST425. Some isolates showed high clonality, despite originating from different hosts. Genes encoding SE or SE-like (SEl) proteins were found in 26 isolates and the toxic shock syndrome toxin encoding *tsst*-1 gene in one. AMR gene carriage was low, though one isolate carried resistance genes to beta-lactams, macrolides, and aminoglycosides.

Wild ungulates may carry enterotoxigenic *S. aureus*. Foodborne illness can be prevented by hygiene and temperature control during processing and storage of game meat, while safe carcass handling can reduce the risk of infection.

## Highlights

• High frequency and large diversity of potentially virulent *S. aureus* in nostrils from wild ungulates in German Federal State.
• Strong increase in *S. aureus* abundance from 2021/22 to 2023/24.
• Low antimicrobial-resistance capacity, but detection of human-adapted strain.
• Possible transmission of *S. aureus* between animals in spatial proximity or common source of colonization.

## 1 Introduction

Compared to livestock and companion animals, data on *Staphylococcus* (*S*.) *aureus* in wildlife remain limited. Nevertheless, *S. aureus* has been detected in a wide range of wild animal species, including rats, hedgehogs, beavers, squirrels, foxes, wild boars, deer, and even bats, elephants, and dolphins [1,2]. In several reviews, the genotypic characteristics of *S. aureus* isolates from wildlife were summarized, revealing a high degree of diversity in terms of sequence types and clonal complexes [1].

*S. aureus* strains from wild animals and game meat have been shown to carry multiple virulence genes. Of particular concern with regard to the pathogenicity of *S. aureus* in general and in wildlife species are the Panton-Valentine leucocidin (PVL) [3,4], the toxic shock syndrome toxin (TSST-1) [5], and staphylococcal enterotoxins (SEs) [6,7]. Producing SEs poses a serious risk in the context of foodborne illness. The classical staphylococcal enterotoxins A to E are most frequently associated with food poisoning [8]. Moreover, the presence of the enterotoxin gene cluster (egc) has been linked to food poisoning [9]. Consuming food contaminated with SEs can result in symptoms such as nausea, vomiting, diarrhea, and abdominal pain. Depending on the virulence capacity, *S. aureus* can cause several illnesses, such as wound infections or mastitis, in a variety of animal species [10]. Furthermore, due to its zoonotic potential, *S. aureus* can be transmitted between animals and humans. While *S. aureus* can harbor numerous virulence-associated genes that, for example, facilitate the adaptation to certain hosts, colonization often occurs without causing clinical symptoms. Its ability to evade the host immune system and produce leucocidins and various toxins makes it one of the most pathogenic bacteria for both humans and animals.

Moreover, antimicrobial resistance (AMR), particularly among methicillin-resistant *S. aureus* (MRSA), is a significant public and veterinary health concern, as treatment options are often limited. MRSA strains are resistant to virtually all beta-lactam antibiotics and frequently exhibit resistance to other antimicrobial classes as well. This resistance is commonly mediated by the *mecA* gene. However, in wildlife species such as hedgehogs, rabbits, and rodents, the *mecC* gene has also been identified [[10], [11], [12], [13]].

Handling wildlife species and their carcasses may lead to transmission of *S. aureus* to humans, increasing the risk of infection or foodborne illness. In addition, transmission of *S. aureus* between wild animals and farm animals may occur due to possible contact between these groups, e. g. when livestock farms are located in close proximity to wildlife habitats.

A longitudinal monitoring of the *S. aureus* occurrence in wild animals intended for human consumption is of high importance for evaluating and updating the risk for hunters, food handlers, and consumers with regard to food safety and human infections. To analyze periodic changes, this study further examines the dynamics of the *S. aureus* occurrence in wild ungulates hunted in the German federal state of Brandenburg in relation to a study conducted two years ago [14].

## 2 Methods

### 2.1 Sample collection and isolation

A framework agreement involving the German Federal Institute for Risk Assessment (BfR) and the German Institute for Federal Real Estate (BImA) permitted sampling activities in the German Federal Forests. Between November 2023 and January 2024, 22 driven hunts were visited in 19 different districts in Brandenburg, Germany. In total, 323 nasal swab samples of wild boars (*n* = 107), roe deer (*n* = 148), fallow deer (*n* = 31), and red deer (*n* = 37) were collected. Samples were collected post mortem within a few hours after death from animals hunted during regular driven hunts in accordance with federal and state hunting and animal protection laws. As no animal was hunted for research purposes, ethical approval was not required. A sampling bag filled with equipment was prepared for every expected hunted animal. In order to avoid cross-contamination during sampling, disposable gloves and hand sanitizer were used. For details regarding the sampling strategy, please refer to Maaz et al. [15]. The samples were transported to the laboratory on the day of collection and stored at 4 °C until further analysis. Swab samples were analyzed for *S. aureus* by selective enrichment using the ISO 6888-3:2003 method [16], which is particularly applicable when low numbers of *S. aureus* are expected. This protocol includes an initial enrichment step in modified Giolitti-Cantoni broth (Merck Millipore, USA) with a 1 % potassium tellurite solution (Merck Millipore, USA) added under anaerobic conditions, followed by subsequent streaking on Baird-Parker agar plates (Oxoid, UK), which include egg yolk tellurite emulsion (Oxoid, UK). According to differences in colony morphology, a loop of one to three presumptive *S. aureus* colonies was transferred to sheep blood agar plates (Oxoid, UK) and incubated for 20–24 h at 37 °C. Species were determined using matrix-assisted laser desorption/ionization time-of-flight mass spectrometry (MALDI-TOF MS; Bruker, USA). Colonies were spotted on the MALDI-TOF-MS target via the direct transfer method and covered with 0.8 μL of α-cyano-4-hydroxycinnamic acid (Bruker Scientific LLC). According to the manufacturer's recommendations, the threshold score for acceptable identification of *S. aureus* species was ≥2.000. The reference database was provided by Bruker (MBT-BDAL-8468).

### 2.2 Whole-genome sequencing and bioinformatic analysis

All isolates identified as *S. aureus* by MALDI-TOF MS were inoculated in 5 mL brain-heart-infusion broth and incubated at 37 °C for 24 h. DNA was extracted from 1 mL culture using the Qiagen DNeasy Blood and Tissue Kit (Qiagen, Germany) according to the manufacturer's protocol modified by adding 10 μL lysostaphin (Sigma Aldrich, USA) to the lysis buffer. The DNA library was prepared using an Illumina DNA Prep kit (Illumina Inc., USA) and the 150 bp paired-end sequencing run was performed on an Illumina NextSeq 500 instrument. Raw Illumina reads were trimmed and de novo assembled using the in-house developed AQUAMIS pipeline [17] (accessed May 2025). Bacterial characterization, such as the determination of sequence types and the analysis of AMR and virulence associated genes, was performed using the in-house developed Bakcharak pipeline (https://gitlab.com/bfr_bioinformatics/bakcharak) (accessed May 2025). Phylogenetic analyses were conducted using core genome multi-locus-sequence-typing (cgMLST) by Ridom SeqSphere+ version 10.5.2 according to the *S. aureus* cgMLST scheme comparing 1861 alleles (accessed May 2025).

### 2.3 Antimicrobial susceptibility testing

Antimicrobial susceptibility testing was performed by broth microdilution according to international guidelines (ISO 20776-1:2006 [18] and CLSI M07, 12th Edition [19]). This involved using a commercial standardized antibiotic panel (Sensititre EUST scheme, Thermo Fisher Scientific, UK), which is recommended by the European Food Safety Authority (EFSA) for resistance monitoring in MRSA from livestock and food [20]. The following EUCAST ECOFFs for *S. aureus* were used for interpretation of the minimum inhibitory concentration (MIC) of the individual isolates: penicillin >0.125 mg/L; cefoxitin >4 mg/L; chloramphenicol >16 mg/L; ciprofloxacin >1 mg/L; clindamycin >0.25 mg/L; erythromycin >1 mg/L; fusidic acid >0.5 mg/L; gentamicin >2 mg/L; kanamycin >8 mg/L; linezolid >4 mg/L; mupirocin >1 mg/L; rifampicin >0.016 mg/L; sulfamethoxazole >128 mg/L; streptomycin >16 mg/L; quinupristin–dalfopristin >1 mg/L; tetracycline >1 mg/L; tiamulin >2 mg/L; trimethoprim >2 mg/L; vancomycin >2 mg/L. For quality control of resistance testing, the *S. aureus* strain ATCC 29213 and *Enterococcus faecalis* strain ATCC 29212 were used.

## 3 Results

### 3.1 Occurrence and genotypic characteristics of *S. aureus* in wild ungulates

*S. aureus* was detected in 14 out of 22 hunting districts in the 2023/24 season with a detection rate per district ranging from 6 % to 67 % (Table 1). The total detection rate of *S. aureus* in nasal swab samples from the hunted wild ungulates was 18 % (58/323). The *S. aureus* detection rate with regard to the wild ungulate species was as follows: Fallow deer (12/31; approx. 39 %), red deer (12/37; approx. 32 %), roe deer (23/148; approx. 16 %) and wild boar (11/107; approx. 10 %). A total of ten different sequence types (STs) were detected for the *S. aureus* isolates (Table 2). Most isolates were associated with ST1 (*n* = 18), ST425 (*n* = 17) and ST133 (*n* = 12). In addition, a few isolates were assigned to ST97 (*n* = 4) and ST3255 (*n* = 2). Moreover, one isolate each was associated with ST30, ST45, ST3224, ST4090 and ST6238.

**Table 1:** Number of nasal swab samples, obtained *S. aureus* isolates and respective detection rate per district in hunting season 2023/24 in Brandenburg, Germany.

District	Samples	Isolates	Detection rate [%]
A	37	11	30
B	15	10	67
C	6	1	17
D	18	1	6
E	20	6	30
F	16	2	13
G	16	3	19
H	23	7	30
J	27	5	19
K	14	2	14
L	21	4	19
M	29	2	7
N	14	1	7
P	13	3	23

### 3.2 Antimicrobial resistance and virulence-associated genes

The number of AMR genes and phenotypic AMR was low (Table 2). One ST30 isolate harboured the β-lactam resistance gene *blaZ*, the macrolide resistance gene *erm*(A), and accordingly showed phenotypic resistance to penicillin (MIC >1 mg/L) as well as erythromycin (MIC >8 mg/L). This isolate additionally carried the *ant*(9)-*Ia* gene, which is responsible for resistance to spectinomycin. Two more isolates also carrying the *blaZ* gene were resistant to penicillin (MIC >1 mg/L). Fifty percent of the isolates (*n* = 29) harboured the *fosB* gene. This gene may be responsible for resistance to fosfomycin. Phenotypic resistance to spectinomycin and fosfomycin were not determined in this study, since these antimicrobials are not part of the used standardized antibiotic panel.

**Table 2:** Detection and number of sequence types (STs), genotypic and phenotypic antimicrobial resistance (AMR) and virulence-associated genes in *S. aureus* isolates in Brandenburg, Germany.

ST	[n]	AMR gene	Phenotypic Resistance	[n]	Virulence-associated genes	[n]
1	18	*blaZ*	Beta-Lactam	3	*seh*	18
30	1	*fosB*	Fosfomycin	29	*sea*	4
45	1	*ant*(9)-Ia	Aminoglycoside	1	*sea*, *seh*	1
97	4	*erm*(A)	Lincosamide/Macrolide	1	*sei*, *sem*, *sen*, *seo*, *seu*	1
133	12				*sea*, *seg*, *sei*, *sem*, *sen*, *seo*, *seu*, *tst*	1
425	17				*sec*, *seg*, *sei*, *sel*, *sem*, *sen*, *seo*, *seu*	1
3224	1					
3255	2					
4090	1					
6238	1					

Virulence-associated genes that are important with respect to food safety and human infections were found in 26 isolates (Table 2). The majority of these isolates harboured the *seh* gene (*n* = 18). Four isolates carried the *sea* gene and one isolate harboured a combination of *sea* and *seh* genes. Moreover, multiple of SE encoding genes were detected in three isolates. One of these additionally carried the toxic shock syndrome toxin encoding *tsst*-1 gene and was associated with ST30.

### 3.3 Phylogenetic relationships

**Fig. 1:**
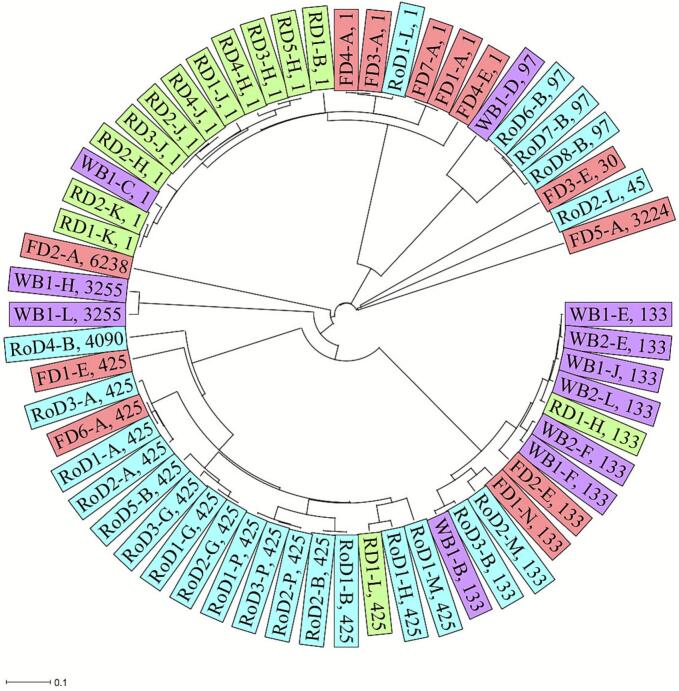
Phylogenetic tree (cgMLST) of *S. aureus* isolates from different wild ungulates species and districts. Wild ungulate species are shown in different colours. Sequence types are represented by numbers. Districts are illustrated by letters A-P. WB = wild boar; FD = fallow deer; RD = red deer; RoD = roe deer. (For interpretation of the references to colour in this figure legend, the reader is referred to the web version of this article.)

**Fig. 2:**
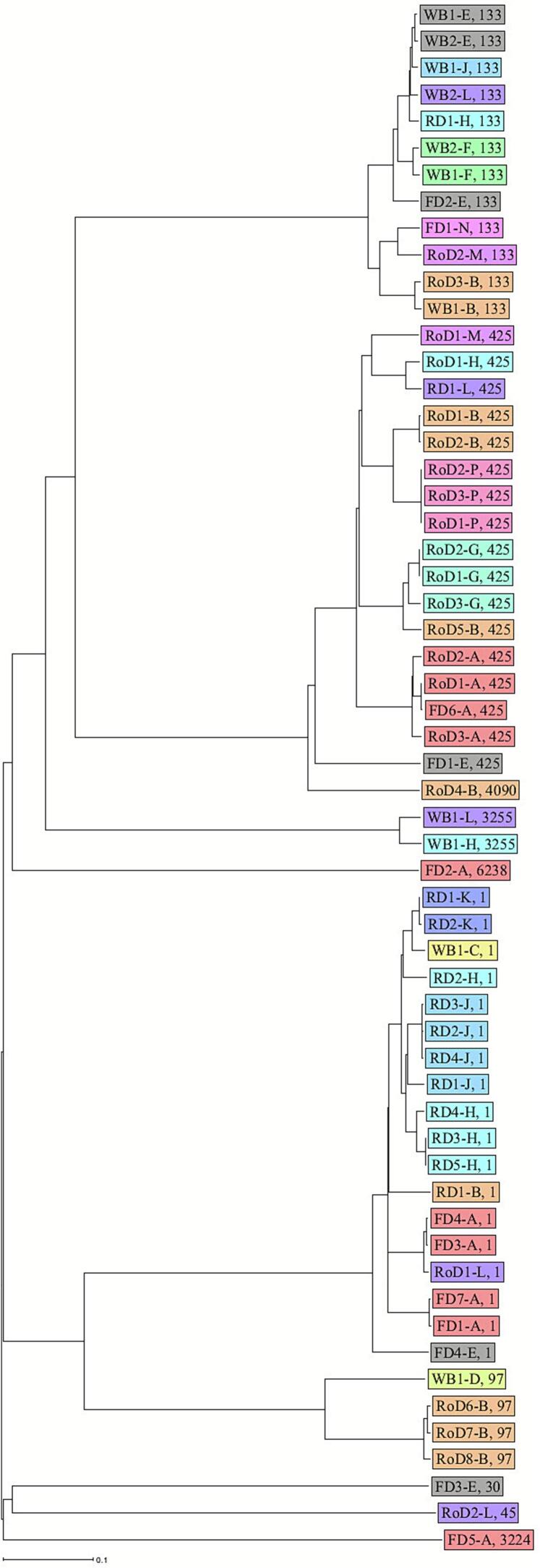
Phylogenetic tree (cgMLST) of *S. aureus* isolates from different wild ungulates species and districts. Districts (A-P) are shown in different colours. Sequence types are represented by numbers. WB = wild boar; FD = fallow deer; RD = red deer; RoD = roe deer. (For interpretation of the references to colour in this figure legend, the reader is referred to the web version of this article.)

The core genomes of the isolates were compared to analyze the phylogenetic relationships. The phylogenetic tree revealed substantial genomic diversity with several clusters of close relationships. ST1 isolates were mainly found in red deer (11/18), whereas the majority of ST425 isolates were from roe deer (14/17) and more than half of the ST133 isolates were from wild boars (7/12) (Fig. 1). An intra-species transmission of *S. aureus* indicated by only few allelic differences was found for several isolates from different wild ungulates from the same hunting district (Fig. 2). Clonal isolates were also detected across different ungulate species from one district, which indicates an inter-species transmission in the same district. In contrast, no close phylogenetic relationships were observed for isolates from different districts.

## 4 Discussion

Monitoring of pathogens in wild animals is important to uncover potential risks for human and livestock health. Pathogens colonizing wildlife species might be transmitted to humans through handling of hunted animals and their carcasses. Moreover, consumers of game meat might be affected by pathogens that produce enterotoxins and thus cause food poisoning. One such pathogen to be monitored is *S. aureus*, which on the one hand may cause severe human infections and on the other hand food poisoning by the production of staphylococcal enterotoxins.

A previous study by the authors analysing samples from the 2021/22 hunting season revealed an *S. aureus* occurrence of 8 % in nasal cavities of hunted wild ungulates in the German federal state of Brandenburg [14]. The study presented here shows the latest numbers from this region and an increase of the *S. aureus* occurrence to 18 % in the 2023/24 hunting season (Table 3). The total number of isolates obtained in the hunting districts increased from 17 in 2021/22 to 58 in 2023/24. Based on the data and knowledge available, it is currently not possible to identify the reason for the increase. The districts from which the wild ungulates originated were identical, and the number of hunts was also comparable to the previous study. The total number of samples collected was higher in the most recent study; however, this probably had only a minor effect on the total detection rate, which is a relative value of isolates with regard to the collected samples. The methodology was identical in both studies, using the ISO 6888-3 isolation method. One reason for higher colonization rates could be a higher wild ungulate population density in the hunting season 2023/24 compared to 2021/22 and thus a greater probability that *S. aureus* is transmitted between animals in close proximity. Since no data on the wild animal population density are available for the respective hunting seasons, the hunting bag may be used as an indirect measure for the population density. However, with regard to data from the Ministry of Agriculture, Food, Environment and Consumer Protection in Brandenburg, the hunting bag of fallow, roe and red deer as well as wild boar in the season 2023/24 was even lower than in 2021/22 [21], so that a higher population density as a reason for an increased *S. aureus* colonization is unlikely. The strong increase of the *S. aureus* occurrence might also include environmental factors such as higher temperatures or increased precipitation in the region in the latest hunting season. The average temperature and total precipitation in Brandenburg in winter 2021/22 were 1.5 °C and 114.8 L/m^2^, respectively, compared to 2.9 °C and 155.2 L/m^2^ in winter 2023/24 [22]. Since the differences in temperature and precipitation between the hunting seasons are only marginal, these factors probably only had a minor effect on the occurrence of *S. aureus*. In addition, the movement pattern as well as the health status of the animals might have played a role with regard to increased colonization rates with *S. aureus*. However, it is very complicated to collect these data, so that it is not possible to conclude about their impact so far.

**Table 3:** Comparison of detection rate and sequence types (ST) of *S. aureus* in wild ungulates in Brandenburg in hunting seasons 2021/22 [14] and 2023/24.

	2021/22	2023/24
Detection rate		
Fallow deer	32 %	39 %
Red deer	13 %	32 %
Roe deer	4 %	16 %
Wild boar	0 %	10 %
Total	8 % [17/215]	18 % [58/323]
Sequence types	S1, ST30, ST133, ST425, ST582, ST6238	ST1, ST30, ST45, ST97, ST133, ST425, ST3224, ST3255, ST4090, ST6238

In this study, an intra-species but also inter-species transmission of *S. aureus* might have contributed to higher colonization rates in the nasal cavities of wild ungulates. As shown by the phylogenetic analyses, closely related isolates were detected in different animals of the same species but also in different wild ungulate species. It can be assumed that direct transmission of these clonal *S. aureus* isolates occurred in close proximity, or that the animals were colonized from a common source, such as surface water, in which *S. aureus* has already been detected in other studies [23]. Interestingly, some *S. aureus* STs were particularly detected in certain wild ungulate species. In general, the majority of *S. aureus* isolates was associated with ST1, ST133 and ST425. It has already been shown that these STs are common in wildlife species [1]. Most of the less frequently found STs (30, 45, 97, 3224, 3255, 4090 and 6238) have also been detected in wild animals before [1], and these STs were also detected in the previous hunting season [14] (Table 3). Previous studies have described an expansion of livestock-associated *S. aureus* to wildlife [24]. In this study, typical livestock-associated *S. aureus* such as ST398, were not detected in wild ungulates; however, ST133 is well-known to be adapted to small ruminants [25]. The presence of the penicillin resistance gene *blaZ* might have been driven by agricultural land cover or livestock farming. A link between land use and antimicrobial resistance in wild ungulates has already been demonstrated in other studies [26,27]. In particular, the *S. aureus* ST30 strain is of high importance for human health, since it is regarded as human-adapted [28]. In this study, the human association of the ST30 isolate was also evident from its carriage of genes encoding resistance to penicillin, erythromycin and spectinomycin. In addition, this isolate harboured genes for staphylococcal enterotoxins and the toxic shock syndrome toxin, which may act as superantigen in the human body and cause severe infections [5].

Staphylococcal enterotoxin genes were present in multiple isolates in this study. The majority carried the *seh* gene, which encodes SEH. Similarly, in the previous study from the same hunting districts, *seh* was the most prevalent SE gene [14]. Although SEH is less potent than the classical SEs, foodborne outbreaks caused by SEH have been reported [29,30]. In total, five isolates carried the *sea* gene, which encodes the classical enterotoxin SEA. SEA is one of the most potent SEs and may cause food poisoning even in low concentrations [8,31,32]. Furthermore, genes of the enterotoxin gene cluster were detected in three isolates. The association between the egc and foodborne outbreaks has already been shown in previous studies [33]. One of the egc-carrying isolates additionally harboured a *sec* gene, which encodes the classical SEC. The production of SEC has also been linked to several foodborne outbreaks [34].

Taken together, the longitudinal monitoring illustrates that wild ungulates in the German federal state of Brandenburg are persistently colonized by *S. aureus* and some of these isolates show a pathogenic potential for human infections and/or food poisoning. The overall occurrence of AMR in the obtained *S. aureus* isolates was very low and comparable to findings from the previous hunting season [14]. Only the *fosB* gene occurred in a relative high number of isolates, which is in line with the previous study from our group [14]. An increasing prevalence of the *fosB* gene in *S. aureus* has also been reported in another study [35]. This suggests that the wild ungulates in this study have only limited contact with livestock farms, where more resistant bacteria can be expected. The circumstances behind the increasing *S. aureus* occurrence in the area are not yet understood, highlighting the importance of ongoing monitoring.

## 5 Conclusion

The colonization rates with potentially virulent *S. aureus* in wild ungulates in Brandenburg, Germany strongly increased with regard to a study conducted two years before. The increasing occurrence of *S. aureus* from 2021/22 to 2023/24 highly demonstrates the importance of further monitoring. Transmission of *S. aureus* may occur between animals in spatial proximity or from a common source of colonization. Handling of hunted animals or their carcasses may contribute to staphylococcal infections in humans. Moreover, food poisoning due to SE-producing strains may occur if recommended hygiene and temperature control practices are not applied during the processing and storage of game meat.

## CRediT authorship contribution statement

**Tobias Lienen:** Writing – review & editing, Writing – original draft, Methodology, Funding acquisition, Formal analysis, Data curation, Conceptualization. **Anneluise Mader:** Writing – review & editing, Writing – original draft, Project administration, Funding acquisition, Data curation, Conceptualization. **Maciej Durkalec:** Writing – review & editing, Writing – original draft, Methodology, Data curation. **Martin H. Richter:** Writing – review & editing, Writing – original draft, Conceptualization. **Sven Maurischat:** Conceptualization, Writing – review & editing, Writing – original draft, Funding acquisition.

## Funding

This study was carried out in the framework of the BfR internal projects No. 1322–769, 44–001 and 8SZ-001.

## Declaration of competing interest

The authors declare that they have no known competing financial interests or personal relationships that could have appeared to influence the work reported in this paper.

## Data Availability

The assembled sequences of all *S. aureus* strains in our study are deposited in NCBI under the BioProject PRJNA634452.
